# Full and partial posttraumatic stress disorders in adults exposed to super typhoon Lekima: a cross-sectional investigation

**DOI:** 10.1186/s12888-021-03528-0

**Published:** 2021-10-18

**Authors:** Rui Zhen, Junjie Zhang, Hongwei Pang, Lingling Ruan, Xuanwen Liu, Xiao Zhou

**Affiliations:** 1 Jing Hengyi School of Education, Hangzhou Normal University, Hangzhou, 311121 China; 2 Teaching and Research Office, Wenling Education Bureau, Wenling, 317500 China; 3 Zhejiang Research Institute of Education Science, Hangzhou, 310028 China; 4 Research and Training Center of School Mental Health Education of Zhejiang Province, Hangzhou, 311121 China; 5 Department of Psychology and Behavioral Sciences, Zhejiang University, Hangzhou, 310028 China

**Keywords:** Super typhoon Lekima, Full PTSD, Partial PTSD, Trauma exposure

## Abstract

**Background:**

Super typhoon Lekima had a maximum wind force of 16 (52 m/s) and hit Wenling city, Zhejiang province in China on August 10, 2019. The typhoon left many victims showing symptoms of posttraumatic stress disorder (PTSD).

**Objective:**

This study aimed to assess the prevalence of full and partial PTSD to inform targeted interventions for adult victims.

**Method:**

In total, four thousand seven hundred and forty-six adults who are parents of students in local primary and middle schools were recruited to participate in this study. Participants completed a trauma exposure scale and the Diagnostic and Statistical Manual of Mental Disorders, Fifth Edition PTSD Checklist. Logistic regression analysis was used to examine the factors of full and partial PTSD.

**Results:**

Nine hundred and ten (19.2%) adults had full PTSD and 1775 (37.4%) had partial PTSD. Adults with a monthly income > 10,000 RMB (about 1530 dollars) and a high education level (bachelor’s degree or above) were less likely to have full or partial PTSD than those with lower income and lower education levels. In addition, married adults were less likely to have full PTSD than divorced or widowed ones. Higher rates of PTSD were observed among those aged ≥40 years, who were injured/trapped, whose family members/friends were injured/trapped, and who lost property.

**Conclusions:**

Partial and full PTSD were common among adults following super typhoon Lekima, and high income, high education level, and married status were protective factors, whereas trauma exposure was a risk factor of PTSD. Target psychological intervention should be provided to these victims who are in low income and education level, divorced and widowed, and experienced more serious trauma.

## Introduction

Super typhoon Lekima had a maximum wind force of 16 (52 m/s) and hit Wenling city, Zhejiang province in China on August 10, 2019. This typhoon affected around 14,024,000 people; 57 people died, 14 were lost, and 2,097,000 required emergency care (Weather China, 2019). Previous studies in different cultures demonstrated that typhoons resulted in loss of life and property, as well as cause various negative psychological outcomes for victims [1–3]. Posttraumatic stress disorder (PTSD) is considered as a common mental disorder following a major typhoon or hurricane [4, 5]. For example, a recent meta-analysis found that the prevalence of PTSD after a hurricane or typhoon was 17.81% [3]. Studies found that PTSD can reduce the marital satisfaction [6], erode the interpersonal relationship [7], increase the risk of suicidal ideation [8] and sleep problems [9]. Thus, it is important to carry out the study in PTSD among people following super typhoon Lekima.

Although previous studies found a high prevalence of PTSD, most studies focused on full PTSD [10, 11] and overlooked the assessment of partial PTSD. A study by Carmassi et al. [12] suggested that meeting the diagnostic criteria of at least one but less than four symptom clusters of PTSD in the Diagnostic and Statistical Manual of Mental Disorders, Fifth Edition (DSM-5 [13];) could be considered as partial PTSD. Therefore, studies have found that victims often reported partial PTSD [10, 11, 14], and showed serious social function problems [15–17]. Assessing the prevalence of PTSD would be helpful to provide target psychological care for the victims following natural disasters.

Full and partial PTSD may be common in victims following natural disasters [17, 18], but not all victims show full or partial PTSD. This raises the question of why some victims report full or partial PTSD whereas others do not. A possible solution is to identify vulnerability markers to enable the prediction of PTSD, which in turn will inform appropriate preventive and treatment strategies for traumatized individuals. Research suggested that predictors of posttraumatic reactions could be categorized into before, within, and post-disaster factors [19, 20]. This study focused on before and within-disaster factors, because these factors can inform individual susceptibility to disasters.

Before-disaster factors include victims’ demographic information [20], such as gender, age, marital status, education level, and family economic status. Previous studies suggested that female gender, older age, being divorced or widowed, and low family economic status were risk factors for posttraumatic reactions following a disaster [21, 22]. Within-disaster factors reflect the severity of trauma exposure [20], which has been found to be associated with PTSD severity [23]. Shattered assumption theory [24] suggested that exposure to a major natural disaster might challenge victims’ positive beliefs about self or the world, or consolidate their negative beliefs about self and the world before trauma. Drawing on the PTSD cognitive model [25], these elicited or strengthened negative views on self and the world may in turn form negative assumptions and elicit negative intrusive thoughts or emotional experiences, which finally result in PTSD [25]. Many studies have shown that degree of trauma exposure was associated with full and partial PTSD [11, 14, 26].

Compared with studies focused on full PTSD, relatively few studies have focused on partial PTSD. Research suggests that some people with partial PTSD may also show serious social function problems [15, 16], meaning it is important to consider partial PTSD in victims following natural disasters. Assessment of predictors of full and partial PTSD along with differentiating protective factors from risk factors will inform targeted psychological services (e.g., family psychological counselling) for relieving the role of risk factors and increase the role of protective factors. This study examined the prevalence of full and partial PTSD in Chinese adults following super typhoon Lekima and assessed predictors of them. In fact, natural disasters have been found to exert negative effects on families [27], leading family members (e.g., parents and children) to develop PTSD. More importantly, parents’ PTSD has an intergenerational effect on children, which may elicits or exacerbates children’ mental problems [28] since children take cues about danger and safety from their parents, and respond to parental distress [29]. Hence, parents are the object of this study. Based on the description above, we hypothesized that female gender, older age, being divorced or widowed, low family economic status, and high trauma exposure were associated with higher levels of full and partial PTSD.

## Methods

### Participants and procedures

Major natural disasters may lead family trauma, in which parents and children show many negative psychological outcomes [30]. In order to relieve family trauma and help family to recover from disasters, we carried out a large-scale psychological investigation in families following natural disasters. The data used in this study is part of this investigation that focused on parents’ psychological adjustment after the super typhoon Lekima. Three months after super typhoon Lekima, we visited Wenling city, Zhejiang province in eastern China. We conducted a large-scale cross-sectional survey and psychological intervention among students and parents to understand the health status of typhoon victims and inform targeted psychological services. With the help of the relevant educational agency and selected school principals, parents of students in grades 4, 5, and 6 in three primary schools and grades 1 and 2 in four middle schools were recruited to answer an online questionnaire packet.

With the help of head teachers in selected classrooms, we sent the online questionnaire link in their parents Wechat (a free messaging and calling app that is widely used in China) group, and explained the purpose of the survey. Some parents might have no time or were not interested in this research, so they did not click the link to fill in the answer at all, but we cannot estimate and monitor these ones through the network. Parents who were willing to fill in the questionnaire carefully completed the questionnaire, ensuring a high response rate. There were 4746 parents participated in our investigation, and 4742 valid questionnaires (response rate 99.9%) were retained after excluding 4 questionnaires that had some indiscriminate answers.

This study was approved by the Research Ethics Committee of the Department of Psychology and Behavioral Sciences, Zhejiang University. The parents of students in selected primary and secondary school were included and there were no exclusion criteria and no compensation was provided to participants. The purpose of the study and voluntary nature of participation were explained to participants before the survey by researchers and the head teachers via WeChat. Online informed consent was obtained from all participants. After completing questionnaire packets, participants were informed that if they needed support, psychological/counseling services were available via a family psychological service website.

### Measures

All of the instruments used in this study were self-report questionnaires. We firstly the prevalence of full and partial PTSD, and then assessed the predictions of demographic variables (e.g., gender, age, monthly income, education level, marital status) and trauma exposure on adult PTSD.

We developed a 10-item scale to assess individuals’ exposure to typhoon based on previous scales bout typhoon and hurricane exposure [1, 31, 32]. The items involved three aspects such as self being injured or trapped, family members or friends being injured or trapped, and loss of property. All items were rated as yes (2 = yes) or no (1 = no).

PTSD was assessed using the revised PTSD Checklist [33] from the Weathers’s [34] PCL-5. This 20-item self-report scale was designed to assess the frequency of PTSD symptoms in relation to the most distressing event experienced by an individual. Participants are asked to rate the frequency of symptom items experienced on a 5-point scale from 0 (not at all/only once) to 4 (almost every week). The scale has four subscales (symptom clusters): intrusions, negative cognition and emotion alteration, avoidance, and hyper-arousal. In this study, the instrument demonstrated good internal consistency (Cronbach’s alpha = 0.96). Cronbach’s alpha for four symptom clusters of PTSD ranged from 0.70 to 0.90. According to the diagnostic criteria of DSM-5 PTSD [13], individuals who simultaneously had at least 1 symptom (score ≥ 2) in the intrusion symptom cluster, 1 symptom (score ≥ 2) in the avoidance symptom cluster, 2 symptoms (score ≥ 2 on each symptom) in the negative alterations in cognitions and mood symptom cluster, and 2 symptoms (score ≥ 2 on each symptom) in the hyper-arousal symptom cluster were classified as full PTSD. That is, meeting the diagnostic criteria of four DSM-5 PTSD’s symptom clusters indicates full PTSD, while meeting the criteria of at least one but less than four symptom clusters indicates partial PTSD [10, 14].

### Data analysis procedure

Statistical analyses were performed using SPSS version 23.0. Frequency analyses, Chi square analyses, and Multivariate logistic regression analyses was respectively used to conduct participants’ demographic characteristics, differences tests, and to examine the roles of demographic variables and trauma exposure in full and partial PTSD. Odds ratios (OR) and 95% confidence intervals (CI) indicated the predictive utility of these factors.

## Results

### Sample characteristics

Of the 4742 participants in this study, 1262 (26.6%) were male and 3480 (73.4%) were female. The mean age was 40.34 years (standard deviation = 5.16 years; range 27–67 years); 1847 (38.9%) were aged < 40 years, 2299 (48.5%) were aged ≥40 years, and 596 (12.6%) did not report their age. In addition, 1710 (36.1%) participants had a monthly income > 10, 000 RMB (about 1530 dollars) and 3032 (63.9%) had a monthly income ≤10, 000 RMB. The majority (*n* = 3789, 79.9%) had a low education level (high school or below) and 953 (20.1%) had a high education level (bachelor’s degree or above). Most (*n* = 4270, 90.0%) were married and 472 (10%) were divorced or widowed. Overall, 852 (18.0%) adults were injured or trapped during the typhoon, 1519 (32.0%) reported injured or trapped family members and friends, and 3466 (73.1%) reported the loss of family property.

### Prevalence of full and partial PTSD

Based on the diagnostic criteria of full and partial PTSD, we found that 910 (19.2%) adults had full PTSD and 1775 (37.4%) had partial PTSD. Table 1 shows the prevalence and the chi-square tests of full and partial PTSD among adults by demographic characteristics and typhoon exposure. We found that the prevalence of full PTSD was higher among adults with age ≥ 40 years, monthly income ≤10, 000 RMB, divorced or single status, and low education level than those with age < 40 years, monthly income > 10, 000 RMB, married status, and high education level. The prevalence of partial PTSD was higher among adults with low education level than those with high education level. The prevalence of both full and partial PTSD were higher among adults who were injured or trapped, whose family members and friends were injured or trapped, and who experienced the loss of property than those who did not have such experiences. In addition, there were no significant gender differences in the prevalence of full and partial PTSD. Partial PTSD had no significant differences in age, monthly income, and marital status.

**Table 1:** Prevalence of full and partial PTSD among adults by demographic characteristics and typhoon exposure

	Full PTSD		Partial PTSD	
	%	χ^2^	%	χ^2^
Gender		1.97		0.50
*Male*	20.5		36.6	
*Female*	18.7		37.7	
Age		12.36^***^		0.76
*≥ 40 years*	21.2		38.4	
*< 40 years*	16.9		37.1	
Monthly income		72.87^***^		3.30
*> 10, 000 RMB (about 1530 dollars)*	12.7		35.7	
*≤ 10, 000 RMB*	22.9		38.4	
Education level		106.01^***^		17.4^***^
*High school or below (low education level)*	22.1		38.9	
*Bachelor’s degree or above (high education level)*	7.5		31.6	
Marital status		19.04^***^		0.14
*Married*	18.4		37.5	
*Divorced or widowed*	26.7		37.4	
Self was injured or trapped		70.64^***^		4.48^*^
*Yes*	29.5		40.6	
*No*	16.9		36.7	
family members or friends were Injured or trapped		98.38^***^		49.51^***^
*Yes*	27.5		44.6	
*No*	15.3		34.0	
Loss of family property		21.58^***^		60.15^***^
*Yes*	20.8		40.7	
*No*	14.8		28.4	

*Note.* ^*^p < 0.05, ^***^p < 0.001

### Predictors of full and partial PTSD

Multivariate logistic regression analyses were performed to examine associations between participants’ demographic characteristics and trauma exposure and full and partial PTSD. Non-significant associations were found between gender and full and partial PTSD. Other measures were significantly associated with full and partial PTSD (see Table 2). Compared with adults without PTSD symptoms, adults aged ≥40 years were more likely to have full PTSD (OR = 1.31, 95% CI: 1.11 ~ 1.60), adults with a monthly income > 10, 000 RMB were less likely to have full or partial PTSD (OR = 0.58, 95% CI: 0.47 ~ 0.71; OR = 0.79, 95% CI: 0.68 ~ 0.92, respectively), those with a high education level were less likely have full or partial PTSD (OR = 0.22, 95% CI: 0.16 ~ 0.30; OR = 0.52, 95% CI: 0.43 ~ 0.63, respectively), and married adults were less likely to have full PTSD (OR = 0.68, 95% CI: 0.51 ~ 0.91). In addition, the odds of full PTSD were higher in adults who had been injured/trapped (OR = 1.68, 95% CI: 1.33 ~ 2.13), whose family members and friends were injured/trapped (OR = 2.72, 95% CI: 2.22 ~ 3.33), and those who lost property (OR = 1.78, 95% CI: 1.43 ~ 2.21). The odds of partial PTSD were higher in adults whose family members and friends were injured/trapped (OR = 2.21, 95% CI: 1.87 ~ 2.61) and those who lost property (OR = 1.96, 95% CI: 1.66 ~ 2.31). Compared with partial PTSD, the odds of full PTSD were higher in those who had been injured/trapped (OR = 1.56, 95% CI: 1.26 ~ 1.95) and those with injured/trapped family members and friends (OR = 1.23, 95% CI: 1.02 ~ 1.49). However, the odds of full PTSD were lower in those with a monthly income > 10, 000 RMB (OR = 0.73, 95% CI: 0.60 ~ 0.89) and those with a high education level (OR = 0.42, 95% CI: 0.31 ~ 0.58).

**Table 2:** Multivariate logistic regression analysis of participants’ demographic characteristics and trauma exposure with partial and full PTSD

	Full PTSD vs NS	Partial PTSD vs NS	Full vs Partial PTSD
	OR [95%CI]	OR [95%CI]	OR [95%CI]
Gender			
*Female*	1.00	1.00	1.00
*Male*	0.98 [0.80 ~ 1.20]	0.90 [0.76 ~ 1.06]	1.09 [0.89 ~ 1.32]
Age			
*< 40 years*	1.00	1.00	1.00
*≥ 40 years*	1.31 [1.11 ~ 1.60]^**^	1.14 [0.99 ~ 1.32]	1.16 [0.97 ~ 1.39]
Monthly income			
*≤ 10, 000 RMB*	1.00	1.00	1.00
*> 10, 000 RMB*	0.58 [0.47 ~ 0.71]^***^	0.79 [0.68 ~ 0.92]^**^	0.73 [0.60 ~ 0.89]^**^
Educational level			
*High school or below*	1.00	1.00	1.00
*Bachelor’s degree or above*	0.22 [0.16 ~ 0.30]^***^	0.52 [0.43 ~ 0.63]^***^	0.42 [0.31 ~ 0.58]^***^
Marital status			
*Divorced or widowed*	1.00	1.00	1.00
*Married*	0.68 [0.51 ~ 0.91]^**^	0.86 [0.67 ~ 1.10]	0.79 [0.61 ~ 1.04]
Self was injured or trapped			
*No*	1.00	1.00	1.00
*Yes*	1.68 [1.33 ~ 2.13]^***^	1.08 [0.87 ~ 1.32]	1.56 [1.26 ~ 1.95]^***^
Family members or friends were injured or trapped			
*No*	1.00	1.00	1.00
*Yes*	2.72 [2.22 ~ 3.33]^***^	2.21 [1.87 ~ 2.61]^***^	1.23 [1.02 ~ 1.49]^*^
Loss of property			
*No*	1.00	1.00	1.00
*Yes*	1.78 [1.43 ~ 2.21]^***^	1.96 [1.66 ~ 2.31]^***^	0.91 [0.72 ~ 1.14]

Note.^*^p < 0.05, ^**^p < 0.01, ^***^p < 0.001

## Discussion

To our knowledge, this is a first attempt to examine full and partial PTSD in a sample of adults following super typhoon Lekima. The findings suggested that full and partial PTSD were common among adults, which is consistent with previous findings [10, 11, 14]. Moreover, we found that before- and within-typhoon factors exerted influence on the occurrence of full and partial PTSD, wherein high income and education levels, and married status were the protective factors, but low income and education levels, and trauma exposure were the risk factors of PTSD.

To be specific, the results showed that the prevalence of full PTSD was 19.2% and that of partial PTSD was 37.4%. The findings supported previous studies [16, 18] that suggested the existence of partial PTSD in victims following natural disasters in addition to full PTSD. This study also found that victims with full PTSD were less than those with partial PTSD. On the one hand, as the diagnostic criteria of partial PTSD were not as strict as that of full PTSD, as well as the weaker association of partial PTSD with dysfunction [15], more adults were classified as partial PTSD. On the other hand, people in Wenling city have been encountered with some typhoons or storms every year, they might have psychological and material preparation before the typhoon Lekima struck, which protected them from experiencing serious PTSD. Hence, although the typhoon elicited adults’ PTSD, partial PTSD sufferers were more than full PTSD ones [35].

The examination of the predictors of full and partial PTSD showed that a monthly income > 10, 000 RMB and a high education level were associated with a lower likelihood of experiencing full or partial PTSD. According to the conservation of resources theory [36], resources are very important for individuals to maintain mental health. Loss of resources may lead individuals to show negative symptoms after stressful experiences, while gain of resources may buffer psychopathological symptoms. A good economic status and high education level are indicators of individuals’ material and psychosocial resources, respectively. Such resources had made up the loss of resources caused by typhoon, and enhanced individuals’ coping with traumatic experiences and psychological distress. Hence, a good economic and educational status buffered the negative effects of typhoon, and showed protective roles in relieving PTSD [37].

In addition, this study showed that married adults had less full PTSD than divorced or widowed ones. In fact, the married ones would better communicate with their spouse when coping with typhoon, such as disclosing their experiences and emotions. These further increased their mutual understanding [38] and openness, trust, and intimacy in marital relationships [39], thereby developing communal coping with trauma [40] and relieving the severity of PTSD. Besides, marriage in China is the integration of two families, thus married ones have more interpersonal resources and support than divorced or windowed ones. Based on the conservation of resources theory [36], interpersonal resources and support as important psychosocial resources might provide or facilitate the preservation of valued resources, which also helped to relieve psychopathology [41] and show less PTSD.

Additionally, this study found that adults aged ≥40 years were more likely to report PTSD, which was consistent with previous findings that older adults were more susceptible to trauma events compared with younger adults [21]. A possible explanation is that compared with adults < 40 years, those aged ≥40 years in China lived a wearier routine life and bore heavier life burdens (e.g., taking care of their old parents and young children). The trauma brought by the typhoon not only challenged their stable life habits, but also further aggravated their life burdens. Such burdens can lead to them to show more serious PTSD following trauma [42]. Because both female and male adults may have these life burdens, the prevalence of their PTSD did not show significant difference. This is also one possible explanation for the inconsistence between our finding and previous studies [22].

Furthermore, trauma experiences, including the participant or their family members and friends being injured or trapped, along with the loss of property [1, 31, 32], were associated with more PTSD compared with those without such experiences. This finding was concordant with previous findings [1, 43–45]. The shattered assumption theory [24] provides a perspective to understand such finding. In specific, experiencing disasters might challenge individuals’ cognition on self and the world, leading to their sense of incompetence in coping with such trauma experiences and eliciting their feelings of unsafety about the world [46]. Subsequently, trauma survivors would lose a sense of perceived control or predictability in the world, which increase their fear [47] and thus lead to more PTSD [48].

Several limitations that should be noted. First, convenience rather than random sampling was used to recruit participants, which might have led to sample bias. Thus, the generalization of results to other samples should be cautious. Second, although the effects of before and within-disaster factors were examined, post-disaster factors such as social support and cognitive rumination were not considered. Third, this cross-sectional design limits our understanding of the trajectory and causality of adult PTSD after super typhoon, future study adopting a longitudinal design will deepen the findings. Moreover, we only included demographic variables, many other potential factors such as social support, personality traits need further exploration.

Despite these limitations, this study found the prevalence of partial PTSD is higher than that of full PTSD in the adults following typhoon Lekima, which suggested that partial PTSD should be also emphasized after natural disasters. In addition, this study found that higher income, higher education level, and normal married status were protective factors against PTSD, and the severity of trauma exposure was a risk factor of PTSD. These informed that targeted intervention should be carried to adults with lower income, lower education level, divorced or widowed status, and severer trauma exposure, after super typhoon Lekima.

## Data Availability

The datasets used during the current study available from the first author on reasonable request.
